# THE INTERPLAY OF SOCIAL INTERACTION, POSITIVE AFFECT, AND SUBJECTIVE AGE IN OLDER ADULTS

**DOI:** 10.1093/geroni/igae098.2107

**Published:** 2024-12-31

**Authors:** Theresa Pauly

**Affiliations:** Simon Fraser University, Vancouver, British Columbia, Canada

## Abstract

How old people feel compared to their actual age, their so-called “subjective age” (SA), is a central predictor of health and well-being across the life span. Felt age can influence lifestyle choices such as attending a social gathering. On the other hand, spending time with other people can elicit feelings of engagement and positive emotions, which could in turn, make people feel younger. The current study aimed to examine the reciprocal association between everyday time spent in social interaction and subjective age in old age. For this purpose, a sample of 108 older adults aged 65–92 years took part in a daily diary study. Over 14 days, participants reported daily social interaction time, positive affect, and subjective age. Multi-level models showed that previous day social interaction time was related to next day subjective age, whereas previous-day subjective age was not related to next-day social interaction time. In addition, the same-day association between social interaction time and subjective age was fully mediated by positive affect. Findings suggest that social connections might foster a sense of feeling young in older adulthood. Further research in this area may offer valuable insights into developing interventions aimed at enhancing subjective well-being and quality of life among older populations.

